# OsSRO1a Interacts with RNA Binding Domain-Containing Protein (OsRBD1) and Functions in Abiotic Stress Tolerance in Yeast

**DOI:** 10.3389/fpls.2016.00062

**Published:** 2016-02-03

**Authors:** Shweta Sharma, Charanpreet Kaur, Sneh L. Singla-Pareek, Sudhir K. Sopory

**Affiliations:** Plant Molecular Biology Group, International Centre for Genetic Engineering and BiotechnologyNew Delhi, India

**Keywords:** abiotic stress, radical induced cell death, rice, RNA binding domain-containing protein, similar to RCD1

## Abstract

SRO1 is an important regulator of stress and hormonal response in plants and functions by interacting with transcription factors and several other proteins involved in abiotic stress response. In the present study, we report OsRBD1, an RNA binding domain 1**-** containing protein as a novel interacting partner of OsSRO1a from rice. The interaction of OsSRO1a with OsRBD1 was shown in yeast as well as *in planta*. Domain–domain interaction study revealed that C-terminal RST domain of OsSRO1a interacts with the N-terminal RRM1 domain of OsRBD1 protein. Both the proteins were found to co-localize in nucleus. Transcript profiling under different stress conditions revealed co-regulation of *OsSRO1a* and *OsRBD1* expression under some abiotic stress conditions. Further, co-transformation of both *OsSRO1a* and *OsRBD1* in yeast conferred enhanced tolerance toward salinity, osmotic, and methylglyoxal treatments. Our study suggests that the interaction of OsSRO1a with OsRBD1 confers enhanced stress tolerance in yeast and may play an important role under abiotic stress responses in plants.

## Introduction

RCD1 is a nuclear protein that interacts with several transcription factors and other stress responsive proteins and has been shown to protect plants against oxidative damage and other stresses (Belles-Boix et al., 2000; Katiyar-Agarwal et al., 2006; Jaspers et al., 2009, 2010; Vainonen et al., 2012). RCD1 belongs to a plant-specific SIMILAR to RCD1 (SRO) gene family, and was first isolated as a CEO (CLONE EIGHTY-ONE) protein, based on its ability to complement ROS sensitivity in yeast cells (Belles-Boix et al., 2000). The *Arabidopsis* genome encodes one RCD1 and five SROs proteins and the loss-of-function mutation in *RCD1* results in highly pleiotropic phenotypes (Overmyer et al., 2000; Jaspers et al., 2009; Teotia and Lamb, 2009). In *Arabidopsis*, *rcd1* mutant is known to be defective in plant development, but mutant of its closest *Arabidopsis* homolog, *sro1* displays normal development (Jaspers et al., 2009). Notably, *rcd1-sro1* double mutant exhibits severe growth defects, indicating unequal genetic redundancy in RCD1 and SRO1 functions. In plants, a new unified nomenclature system has been proposed for SRO genes since naming conventions based on *Arabidopsis thaliana* were not found to be suitable for most other plant species (Jaspers et al., 2010). However, the previous annotations of *Arabidopsis* SRO genes have been retained in the new nomenclature.

SRO has been classified as a novel sub-family of proteins involved in ubiquitin and ADP-ribose conjugation systems. The members of SRO family contain a conserved globular domain, WWE (named after three of its conserved residues, W and E; PF02825) which is predicted to mediate specific protein–protein interactions (Aravind, 2001) and a region similar to the catalytic domain of poly (ADP-ribose) polymerase proteins (PARP signature; PF00644; Ahlfors et al., 2004) that mediates attachment of ADP-ribose units from NAD^+^ to target proteins and have implications in a number of processes, such as DNA repair, apoptosis, transcription, and chromatin remodeling (Hassa and Hottiger, 2008). The domain composition of SRO proteins is unique within plants as SRO proteins apart from WWE and PARP domains, possess a C-terminal RCD1-SRO-TAF4 domain (RST domain; PF12174) that is believed to be critical for interaction with several, mostly plant-specific, transcription factors (Jaspers et al., 2010). In plants, SRO genes have been classified into two structural types based on their domain composition (Jaspers et al., 2010). The genome of the monocot plants including rice, have been found to contain only group I SRO genes (hence, named as SRO1) which possess all the three domains, viz. WWE, PARP, and RST whereas eudicots in addition to group I SRO genes, contain group II SRO genes (named as SRO2) as well, characterized by the absence of N-terminal WWE domain but having PARP and RST domains. In rice genome annotation database RGAP7, SRO1 is named as Radical induced cell death (RCD1) gene, but as per the nomenclature proposed by Jaspers et al. (2010), we will now refer rice *RCD1* genes as *SRO1*.

The first reported interacting partner of RCD1 protein was STO (salt tolerance; Belles-Boix et al., 2000), a protein that confers salt tolerance to yeast and contains two putative zinc fingers (Lippuner et al., 1996) similar to those found in the transcription regulators, GATA-1 (Putterill et al., 1995) and CONSTANS (Orkin, 1996). The other RCD1 interacting partner includes a putative protein, similar to the members from the Ethylene Responsive Element Binding Protein (EREBP) sub-family of AP2/EREBP plant transcription factors. Members of this protein sub-family are involved in the induction of defense genes in response to biotic and abiotic stresses (Zhou et al., 1997; Fujimoto et al., 2000). Further, an interaction between the predicted cytoplasmic tail of SOS1, a sodium transporter, and RCD1 has been proposed in *A. thaliana* (Katiyar-Agarwal et al., 2006) and its interaction with DREB2A is also reported (Belles-Boix et al., 2000; Vainonen et al., 2012), which reveals a function for RCD1 in stress tolerance. In *Arabidopsis*, it has been shown that loss of *rcd1* expression leads to malfunctioned control of cell death in response to apoplastic ROS and that *WRKY70* and *SGT1b* work as cell death regulators downstream of RCD1 (Brosché et al., 2014). Further another study reports that RCD1 protects plant cells from activating ROS-triggered programs, such as cell death and induction of pathogen-responsive genes (PR genes) and extra-plastidic antioxidant enzymes, by supporting the induction of chloroplast antioxidant system via interactions with the transcription factor Rap2.4a (Hiltscher et al., 2014). In rice, a member of SRO family, *OsSRO1c*, is reported to play an important role in drought and oxidative stress tolerance of rice by promoting stomatal closure and H_2_O_2_ accumulation through a novel pathway involving SNAC1 (stress-responsive NAC 1) and a zinc-finger gene, DST (You et al., 2013).

The rice SRO gene family consists of five members named as OsSRO1a, OsSRO1b, OsSRO1c, OsSRO1d, and OsSRO1e. In the present study, we have identified interacting partners of rice OsSRO1a. Our results show that OsSRO1a interacts with several proteins. Of these, we have characterized a novel interacting partner, the RNA binding domain (RBD) containing protein, OsRBD1. Detailed studies suggest that both the proteins are localized in nucleus and their interaction confers stress tolerance in yeast. Altogether, our results suggest that this interaction may have relevance in stress physiology.

## Materials and Methods

### Genomic Distribution of OsSRO Genes on Rice Chromosomes

Position of each of the *OsSRO* genes on rice chromosome available at RGAP version 7 was determined^1^ and multiple alignment of all the isoforms of rice SRO1 proteins was performed using ClustalW2 (Larkin et al., 2007).

### Expression Analysis Using Rice Genome Database

To analyze the expression of *OsSRO1* genes *in silico*, we used Gene Expression Omnibus platform^2^ accession number GSE6893 and GSE6901 for reproductive development and stress response, respectively. Expression data was further depicted by heatmap generated with the help of MeV software package.

### Plant Material, Growth Conditions, and Sample Collection

Seedlings of IR64 rice cultivar were grown under controlled conditions in growth chamber at 28 ± 2°C and 16 h light/8 h dark photoperiod. After sterilization with 1% Bavistin for 20 min, seeds were germinated hydroponically in modified Yoshida et al. (1972) medium. Various treatments, including low and high temperature (4 and 42°C, respectively), desiccation (air dry), salinity (200 mM NaCl), MG (5 mM), oxidative (5 mM H_2_O_2_) and wounding (pricking the leaf with a needle) were given to the 12 days-old seedlings and shoot tissue was harvested after 6 h and 24 h of stress treatment. The shoots of untreated seedlings served as control.

### Cloning of OsSRO1a and OsRBD1 from Rice

Os*SRO1a* and Os*RBD1* were amplified from rice cDNA using gene specific primers (**Supplementary Table S1**) and cloned into TOPO-TA vector (Invitrogen, USA). For localization assay, each of the *OsSRO1a* and *OsRBD1* cDNA were cloned in pMBPII vector as done previously (Kumar et al., 2012) at BamH1 and XbaI/BamH1 sites, respectively. For *in planta* interaction studies, *OsSRO1a* and *OsRBD1* cDNA were cloned in BiFC1 and BiFC2 vectors at Not1 and Nco1/Not1 sites, respectively. *OsSRO1a* and *OsRBD1* full length cDNA were also cloned in pYES2 yeast expression vector (Invitrogen, USA) at BamH1 and HindIII/BamH1 for yeast expression studies.

### Yeast Two Hybrid Assay (YTH) for Identification of Interacting Partners in Rice Library

A Gal4-based two-hybrid system (Clontech, USA) was used for YTH assay as described by Kumar et al. (2012). The pGAD plasmid which contains the DNA activation domain of Gal4 (AD) and/or pGBD which contains the DNA binding domain of Gal4 (BD) were used to express the AD and BD fusion proteins, respectively. In brief, *OsSRO1a* encoding the bait protein was ligated into the pGBD vector using EcoRl/Sal1 sites. AH109 strain (*MAT*a *trp1-901 leu2-3, 112 ura3-52 his3-200 gal4*Δ *gal80*Δ *LYS2::GAL1_UAS_-GAL1_TATA_-HIS3 GAL2_UAS_-GAL2_TATA_-ADE2 URA3::MEL1_UAS_ -MEL1_TATA_ -lacZ*) was used for transformation. Competent cells were made from glycerol stocks of the rice cDNA library (already cloned in pGAD vector), followed by its transformation with the bait plasmid. The transformants were grown on the two drop out (-Leu-Trp-) and three drop-out (-Leu-Trp-His) medium, followed by four drop-out (-Leu-Trp-His-Ade) medium supplied with 20 mM 3-AT (3-amino-1,2,4-Triazole) to find out the interacting partners.

Yeast monohybrid assay was performed by co-transforming OsSRO1a-pGAD and pGBD vector or co-transforming OsSRO1a-pGBD and pGAD vector followed by assessing the growth of transformants on two drop out (-Leu-Trp-) medium to confirm that if is any self interaction.

### Construction of Deletion Mutants of OsSRO1a and OsRBD1

Deletion mutants for *OsSRO1a* and *OsRBD1* were made to investigate domain to domain interactions. For this purpose, a set of six primers were designed to differentially amplify different domains (**Supplementary Table S1**). For *OsSRO1a*, three sets of primers were designed which were used for amplification of partial fragments of *OsSRO1a*, such that first fragment contained N-terminal (OsSRO1a_N), second contained PARP domain (OsSRO1a_PARP), while the third fragment contained RST domain (OsSRO1a_C). Primers for *OsRBD1* deletion studies were also designed in a similar way so as to amplify different regions of the gene in three fragments (**Supplementary Table S1**). First fragment contained N-terminal region (OsRBD1_N), second containing RRM1 domain [OsRBD1_RRM1; RNA recognition motif (RRM) domain] and third containing C-terminus (OsRBD1_C). For checking one to one interaction between *OsSRO1a* and *OsRBD1*, both the genes were cloned in frame into both pGAD and pGBD vectors, resulting in various combinations of constructs.

### Protoplast Isolation and Subcellular Localization

Protoplast isolation and transfection was performed following the method of Sheen (2001). In brief, soft and young leaves from 25 days-old tobacco plants were chopped finely and lysed with enzyme solution containing 1% cellulase Y-C, 0.1% macerozyme, (Kyowa Chemical Products, Osaka, Japan), 0.4 M mannitol, 5 mM MES, pH 5.7, filter sterilized. The mixture was incubated in a small petridish at 25°C for 3 h in dark and centrifuged at low speed (145 g, Eppendorf 5810R) followed by washing with wash buffer (0.4 M mannitol, 2.5 mM CaCl_2_, 1 mM MES, pH 5.7) and finally, the protoplasts were resuspended in 20 ml solution containing 0.4 m mannitol, 15 mM MgCl_2_, 5 mM MES, pH 5.7. The protoplasts were then diluted in a solution containing 154 mM NaCl, 125 mM CaCl_2_ 5.0 mM KCl, 2.0 mM MES (pH 5.0) and 2.0 μg of OsSRO1a-pMBPII and OsRBD1-pMBPII plasmids were added along with 40% PEG and mixed gently. The mixture was then incubated for 20 h at 23°C with gentle agitation. The incubation buffer was subsequently removed and protoplasts were viewed via fluorescence microscopy (Zeiss observer Z1).

### Particle Bombardment and Fluorescence Microscopy

Onion peel bombardment assay was performed as described previously (Kaur et al., 2014). About 3 μg of OsSRO1a-pMBPII and OsRBD1-pMBPII plasmids were coated separately on 1 μm gold particles and introduced into onion epidermal cells by microprojectile bombardment, using a Bio-Rad PDS/1000 helium-driven particle accelerator, as per the manufacturer’s instructions. Plates were incubated at 28°C for 18 h in dark before microscopic analysis. Transformed epidermal onion peels were observed under fluorescence microscope (Zeiss observer Z1). For nucleus staining, onion peels were briefly incubated with 100 nM DAPI (4, 6-diamidino-2-phenylindole, dihydrochloride) stain (Invitrogen, USA), prior to microscopic analysis.

### Bimolecular Fluorescence Complementation Analysis

To confirm interactions *in planta*, *OsSRO1a* and *OsRBD1* were cloned in BiFC1 and BiFC2 vectors, respectively and transformed into *Agrobacterium* (LBA4404). Empty vectors were also transformed as controls. All the constructs were then inoculated in YPD media (containing yeast extract, peptone, and dextrose) till absorbance reached 1.2 and the cells were collected by centrifugation at 5000 g. Cultures were then resuspended in 10 mM of MgCl_2_ and 10 mM MES containing buffer and incubated for 4 h at 28°C. Cell cultures containing *OsSRO1a* and *OsRBD1* constructs were mixed and agro-infiltrated into 25 days-old tobacco leaves. Both the empty vectors were also pooled together and infiltrated as control.

### Real Time PCR

Real Time PCR was performed as described previously (Mustafiz et al., 2011). Total RNA was isolated from the shoot tissues of control and stressed seedlings using RaFlex^TM^ solution I and solution II (GeNei, India), and cDNA synthesis was performed with RevertAid^TM^ RNAse H minus cDNA synthesis kit (Fermentas Life Sciences, USA). Manufacturer’s protocol was strictly followed in both the above procedures. Real time PCR primers for *OsSRO1a* and *OsRBD1* were designed from 3′ UTR regions using Primer3 software (see **Supplementary Table S2** for primer sequences). Three replicates of each sample (control and stress treated) were used. Mean Ct values were calculated for each sample and further normalized against the maximum expression value obtained for the control sample (Livak and Schmittgen, 2001). *eEF*-*1α* gene was used as internal control. The statistical significance of stress-induced change in *OsSRO1a* and *OsRBD1* transcript levels at each time point compared to control was tested by paired *t*-test (one-sided) using Q-Gene (Simon, 2003).

### Stress Tolerance Assay for the Significance of Interaction Between OsSRO1 and OsRBD1

*OsSRO1a* and *OsRBD1* cloned in pYES2 vectors were transformed, individually as well as together in yeast BY4741 (*MAT*a *his31Δ leu2*Δ*1 met 15*Δ*0 ura3*Δ0) strain. Transformed strains were grown overnight in YPD medium at 30°C and diluted to 0.5 OD_600_. Serial dilutions (1:10, 1:100, 1:1000, and 1:10,000) of 0.5 OD_600_ were then spotted on solid YPD medium supplemented with various stress-inducers to study the significance of OsSRO1a and OsRBD1 interaction in response to salinity (1.2 M NaCl), oxidative (10 mM H_2_O_2_), osmotic (1 M mannitol), and methylglyoxal (8 mM) treatments. Growth pattern for each of the yeast transformants was also observed by streaking assay on stress and control media Empty vector was also transformed and used as control.

## Results

### Identification and Chromosomal Localization of OsSRO Genes

**FIGURE 1:**
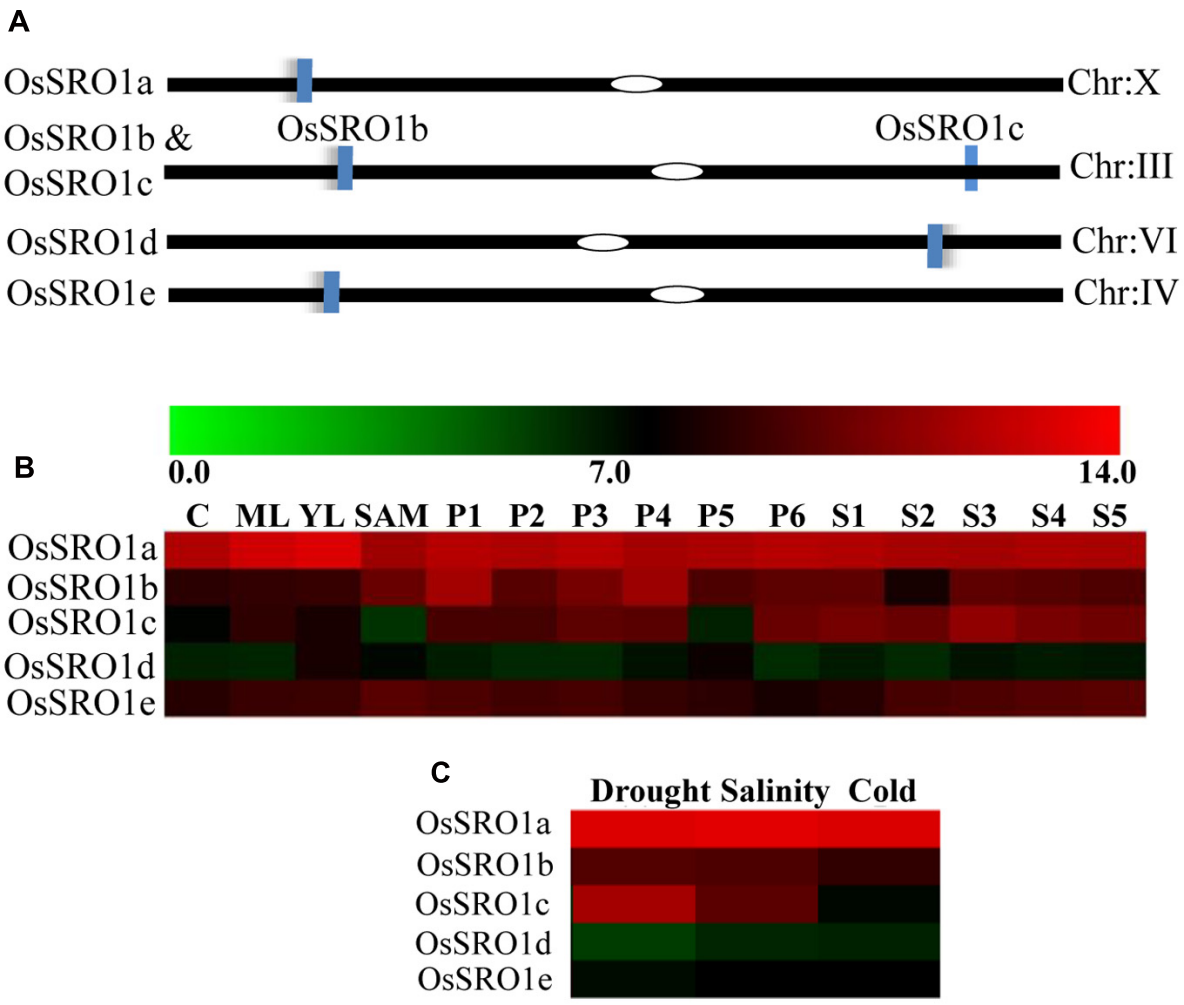
**Rice SRO1 genes and their genomic distribution on chromosomes.** **(A)** Genomic distribution of *OsSRO1* genes on rice chromosomes. Chromosome number is indicated at the right and blue bars represent position of the *OsSRO1* genes. **(B)** Expression profile of *OsSRO1* genes at different developmental stages as mentioned on top of each column viz. 7 days seedlings taken as control (C), mature leaf (ML), young leaf (YL), shoot apical meristem (SAM), young inflorescence (PI, up to 3 cm), inflorescence (P2, 3–5 cm; P3, 5–10 cm; P4, 10–15 cm; P5, 15–22 cm; P6, 22–30 cm) and seed (S1, 0–2 dap; S2, 3–4 dap; S3, 5–10 dap; S4, 11–20 dap; S5, 21–29 dap), and **(C)** stress conditions such as drought, salinity, and cold using microarray data. Color bar at the top represents expression values, thereby green color representing lowest expression levels and red signifying highest expression level.

Five *OsSRO* genes were identified from the RGAP 7 database, distributed on chromosome X, III, VI and IV, and named as OsSRO1a, OsSRO1b, OsSRO1c, OsSRO1d, and OsSRO1e, respectively (**Figure 1A**) with both OsSRO1b and OsSRO1c located on the chromosome III. Multiple alignments of the amino acid sequences of all the five SRO1 proteins revealed about 40–61% similarity among them. OsSRO1a was most similar to OsSRO1b sharing 61% homology in amino acid sequence, followed by OsSRO1e possessing 49% sequence similarity. Domain search analysis^3^ showed that all the OsSRO1 proteins contain an N-terminal WWE domain, a catalytic core, comprising poly (ADP-ribose) polymerase (PARP) domain and a C-terminal RST (RCD1-SRO-TAF4) domain.

### Developmental and Stress-Specific Regulation of Rice SRO1 Genes

To examine the expression profile of rice *SRO1* genes at various stages of development and under various abiotic stress conditions, publicly available microarray data^4^ was analyzed. Analysis of microarray dataset indicated that *OsSRO1a, OsSRO1b, and OsSRO1e* genes expressed constitutively across various developmental stages, as compared to *OsSRO1c* and *OsSRO1d* (**Figure 1B**). In response to drought, cold and salt stress, *OsSRO1a* was significantly up-regulated in comparison to other *OsSRO1* genes (**Figure 1C**). Thus, being highly stress-inducible, *OsSRO1a* was selected for detailed studies to identify its role in abiotic stress response.

### Identification of Interacting Partners of OsSRO1a

Domain analysis revealed the presence of a typical RST domain in OsSRO1a at its C-terminus, which suggests that OsSRO1a may associate with other proteins to elicit its regulatory roles. In an attempt to identify the potential interacting partners, full-length ORF of *OsSRO1a* (LOC_Os10g42710) was amplified and cloned. Yeast two hybrid assay (YTH) was performed using OsSRO1a-pGBD construct as bait protein, and yeast library of rice as prey. A number of clones grew on four drop-out medium (Trp^-^ His^-^ Ade^-^ Leu^-^) and further confirmed by transferring the colonies to medium containing increasing concentration (10, 15, and 20 mM, respectively) of 3-amino-1,2,4-triazole (3AT). Even in the presence of 20 mM 3AT, a large number of colonies appeared. In order to reconfirm if this was not due to trans-activation, monohybrid assays were performed which suggested that OsSRO1a does not act as a trans-activator (data not shown). Most of the colonies that grew were found to be positive, with varying length of inserts. Plasmid from about 100 colonies was isolated, sequenced and analyzed using NCBI-BLAST search, which revealed that OsSRO1a may interact with several proteins such as TBC domain-containing protein, RRM containing protein, CAX-interacting protein, universal stress protein domain containing protein and many more (**Supplementary Table S2**). Among all the interacting partners, OsRBD1 was found to be predominantly present and hence selected for further studies. The *OsRBD1* gene (LOC_Os12g01010) is located on chromosome number 12 and domain analysis using Pfam showed the presence of RRM1 at its N-terminus. Sequence analysis of *OsRBD1* using BindN tool^5^ predicted strong binding sites for RNA where about 84 out of 227 residues were predicted to be RNA binding amino acids.

### RST Domain of OsSRO1a Interacts with the RRM1 Domain of OsRBD1

**FIGURE 2:**
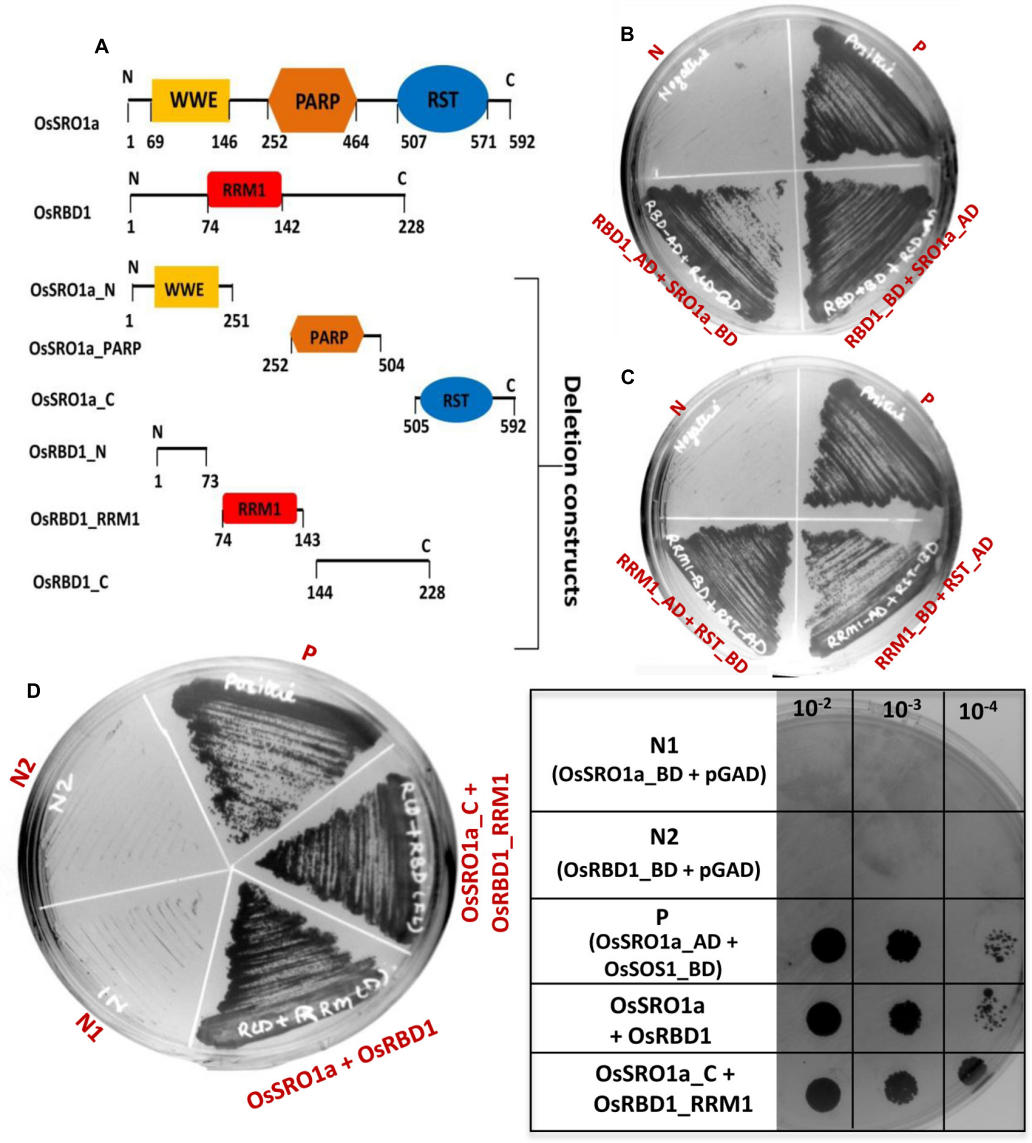
**RST domain of OsSRO1a interacts with RRM1 domain of OsRBD1.** **(A)** Schematic representation of the domains present in OsSRO1a and OsRBD1 proteins and various deletions constructs prepared for domain–domain interaction studies. **(B)** Growth observed after co-transforming full-length OsRBD1 (in pGAD vector) and OsSRO1a (in pGBD vector), and OsRBD1 (in pGBD vector) and OsSRO1a (in pGAD vector), and **(C)** after co-transforming OsRBD1_RRM1 (in pGAD vector) and OsSRO1a_RST (in pGBD vector), and OsRBD1_RRM1 (in pGBD vector) and OsSRO1a_RST (in pGAD vector) on four drop-out + 3-AT (20 mM) medium. **(D)** Growth observed after streaking co-transformed full-length OsRBD1 and OsSRO1a (OsSRO1a + OsRBD1) and OsSRO1a_C and OsRBD1_RRM1 (SRO1_C + RRM1) on four drop-out + 20 mM 3-AT medium (left panel) and spotting serial dilutions of the same (right panel). P is the positive control taken as OsSRO1a-pGAD + OsSOS1-pGBD, and N, N1, and N2 are the negative controls, representing co-transformed empty vectors, OsSRO1a_BD + pGAD and OsRBD1-BD + pGAD, respectively. The data shown is representative of three clones used for each construct and experiment was repeated thrice.

To study specific domain–domain interactions between OsSRO1a and OsRBD1, different deletion constructs were designed. Based on the domain architecture of OsSRO1a and OsRBD1 proteins, different deletion fragments were prepared, selectively amplifying different domains (**Figure 2A**). Firstly, a protein to protein interaction study was carried out using constructs containing full-length *OsSRO1a* and *OsRBD1* proteins as bait and prey, respectively. Colonies co-transformed with *OsSRO1a* and *OsRBD1* constructs could grow well on four drop-out + 3-AT medium (**Figure 2B**). Next, domain wise interactions were studied using constructs containing different deletion fragments of *OsSRO1a* and *OsRBD1* in different combinations. Out of all combinations tested, OsRBD1_RRM1 and OsSRO1a_C could successfully grow on four drop-out + 3-AT medium (**Figure 2C**). The growth of other co-transformants was severely hampered even in the three drop-out medium (data not shown). For further confirmation, full-length and deletion constructs which could grow well on four drop-out medium were then spotted on four drop-out + 3-AT (20 mM) medium (**Figure 2D**). The combination of OsSRO1a-pGAD + OsSOS1-pGBD constructs was used as a positive control. N, N1, and N2 indicate negative controls which represented co-transformed empty vectors, OsSRO1a_BD + pGAD and OsRBD1-BD + pGAD constructs, respectively. The data clearly indicated that the RST domain at the C-terminus of OsSRO1a (OsSRO1a_C) interacts with the RRM1 domain at N-terminus of OsRBD1 (OsRBD1_RRM1) protein. The summary of interactions between OsSRO1a and OsRBD1 proteins is shown in **Table 1**.

**Table 1:** Summary of interaction between different domains of OsSRO1a and OsRBD1.

OsSRO1a	OsRBD1	Interaction
Full length (OsSRO1a)	Full length (OsRBD1)	+++
OsSRO1a_N	Full length (OsRBD1)	---
OsSRO1a_PARP	Full length (OsRBD1)	---
OsSRO1a_C	Full length (OsRBD1)	+++
Full length (OsSRO1a)	OsRBD1_N	---
Full length (OsSRO1a)	OsRBD1_RRM1	+++
Full length (OsSRO1a)	OsRBD1_C	---
OsSRO1a_C	OsRBD1_RRM1	+++


### Subcellular Localization of OsSRO1a and OsRBD1

**FIGURE 3:**
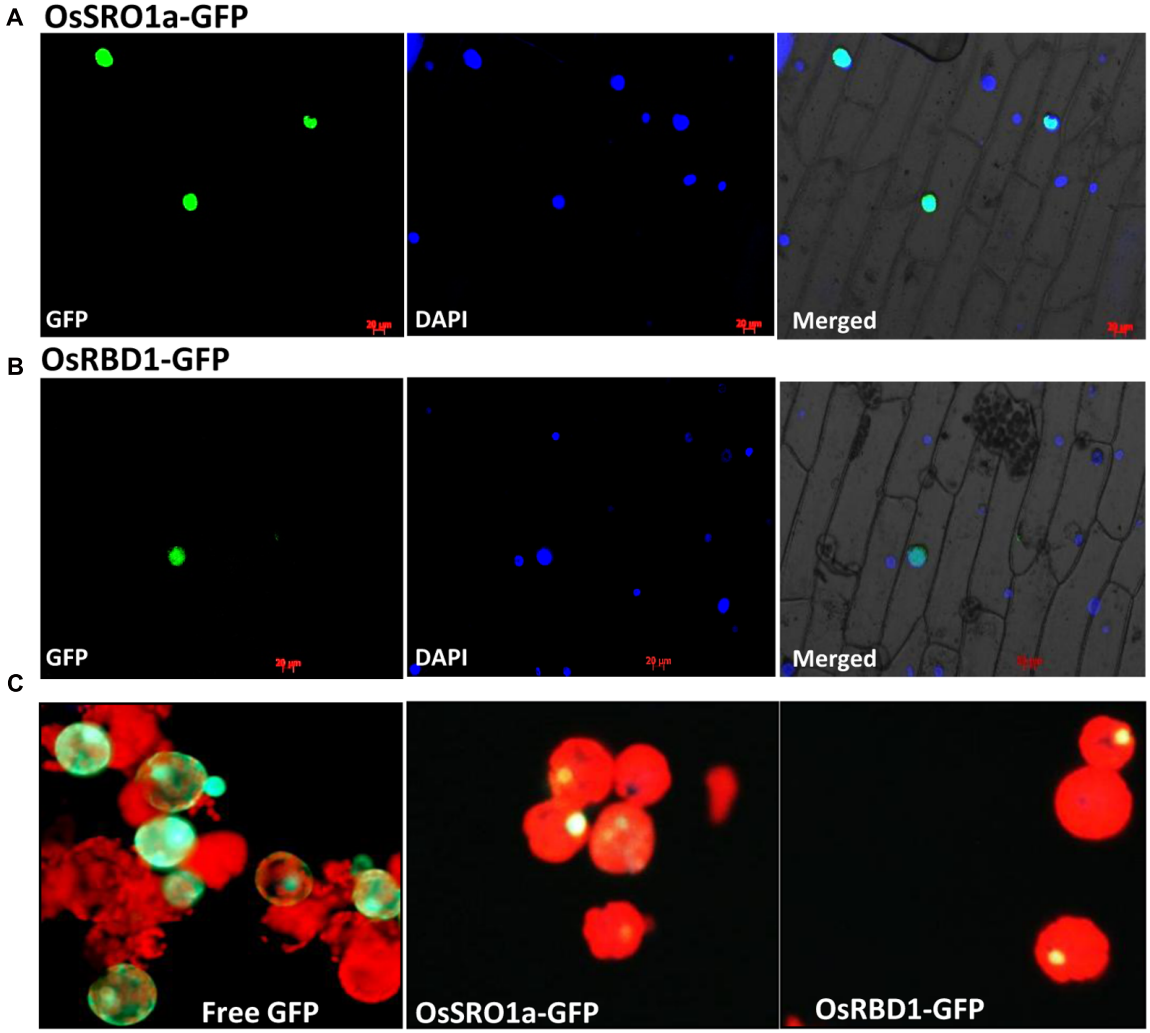
***In vivo* localization of OsSRO1a and OsRBD1.** Onion peel epidermal cells showing expression of **(A)** OsSRO1a-GFP and **(B)** OsRBD1-GFP driven by CaMV 35S promoter in the nucleus, as indicated by the superposition of GFP and DAPI dye fluorescence. **(C)** Tobacco leaf protoplasts transfected with pMBPII vector alone (for free GFP visualization; left panel) and, OsSRO1a-pMBPII (middle panel) and OsRBD1-pMBPII (right panel) to confirm the nuclear localization of OsSRO1a and OsRBD1, respectively.

For localization studies, *OsSRO1a* and *OsRBD1* cDNAs were amplified and cloned into pMBPII vector in translational fusion with GFP at the C-terminus. The resulting pMBPII-*OsSRO1a* and pMBPII-*OsRBD1* constructs were used for particle bombardment in onion peel epidermal cells. Both *OsSRO1a* and *OsRBD1* were found to be localized in nucleus, as confirmed by superposition of fluorescence image of the nucleus staining dye DAPI with that of GFP (**Figures 3A,B**)

Localization of *OsSRO1a* and *OsRBD1* was also analyzed in tobacco protoplasts. Transiently transfected protoplasts with vector alone (pMBPII) showed green fluorescence of free GFP throughout the protoplasts (**Figure 3C**). However, transfection with either OsSRO1a-GFP or OsRBD1-GFP showed green fluorescence restricted to nucleus only thereby, indicating nuclear localization of OsSRO1a and OsRBD1 (**Figure 3C**).

### *In planta* Interaction of OsSRO1a and OsRBD1

**FIGURE 4:**
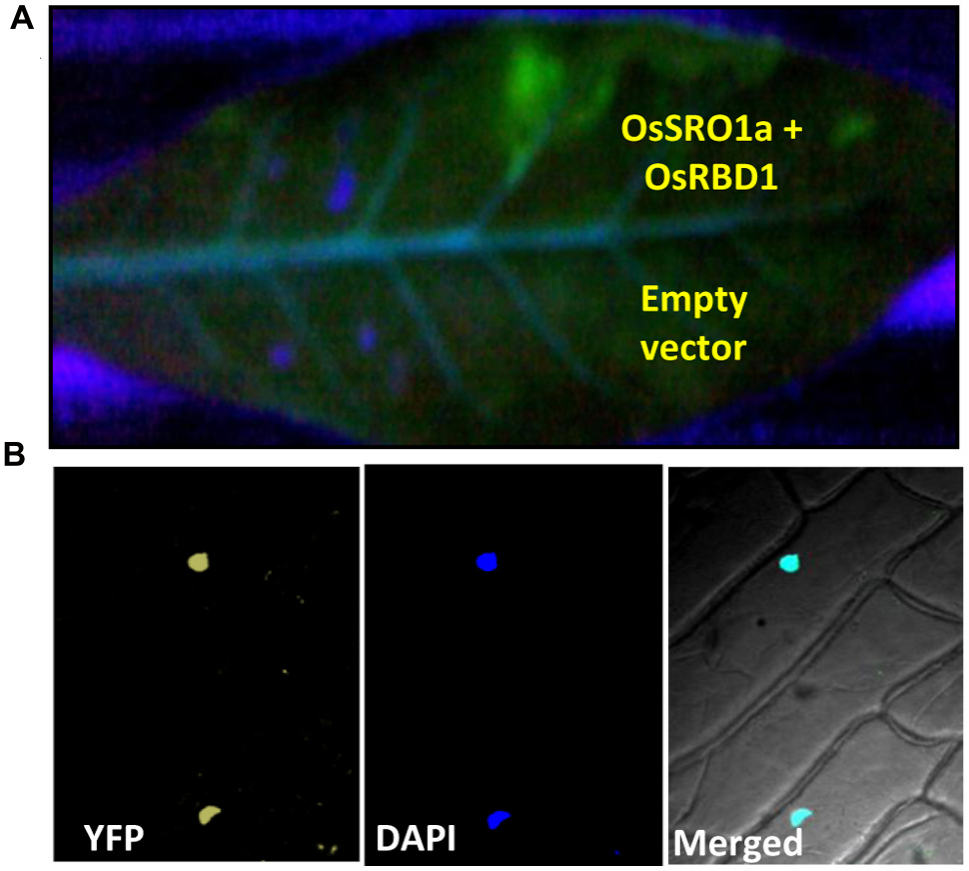
**Confirmation of OsSRO1a and OsRBD1 interaction *in planta*.** **(A)** *Agroinfiltration* of OsSRO1a-BiFC1 + OsRBD1-BiFC2 constructs in *Nicotiana tabacum* leaves. Upper side of the midrib shows co-infiltration of OsSRO1a-BiFC1 and OsRBD1-BiFC2 constructs and the lower side shows co-infiltration of BiFC1 and BiFC2 empty vectors. **(B)** Onion-peel bombardment assay to confirm *in vivo* interaction between OsRBD1-BiFC1 and OsSRO1a-BiFC2.

The bimolecular fluorescence complementation (BiFC) assay is based on the observation that the association of fluorescent protein fragments can be facilitated by an interaction between proteins fused to the fragments in a split YFP system. In principle, the BiFC assay can be used to visualize interactions between any proteins that can be fused to fluorescent protein fragments. To confirm the interaction *in planta*, both the interacting partners were cloned in BiFC vectors resulting in *OsSRO1a*-BiFC1 and *OsRBD1*-BiFC2 constructs. Recombinant *OsSRO1a*-BiFC1 and *OsRBD1*-BiFC2 constructs were transformed in *Agrobacterium* strain and positive clones were selected for further studies. Before infiltration, culture of *Agrobacterium* cells containing *OsSRO1a*-BiFC1 and *OsRBD1*-BiFC2 plasmids were mixed and then infiltrated into tobacco leaf. Different constructs, including recombinant plasmids and control, were infiltrated in the same leaf to avoid any artifact due to the age or position of the leaf. Suspensions of *A. tumefaciens* carrying both the constructs were infiltrated into the left half of the leaf and agro-suspension containing empty vectors were also mixed and infiltrated on the right side of the leaf. Highly fluorescent left half of the leaf was observed under UV after 24 h of infiltration while there was no fluorescence on the right side where empty vectors were infiltrated even when checked at 36 and 48 h. This showed that a strong interaction occurs between OsSRO1a and OsRBD1 proteins *in planta* (**Figure 4A**).

To reconfirm *in vivo* OsSRO1a-OsRBD1 interactions, *OsSRO1a*-BiFC1 and *OsRBD1*-BiFC2 constructs were also transformed in onion peel epidermal cells. When viewed under fluorescence microscope, bombarded peels containing both the proteins showed yellow fluorescence exclusively in nucleus while no fluorescence was detected where empty vectors were bombarded (**Figure 4B**).

### Expression Analysis of OsSRO1a and OsRBD1 in Response to Different Abiotic Stresses

**FIGURE 5:**
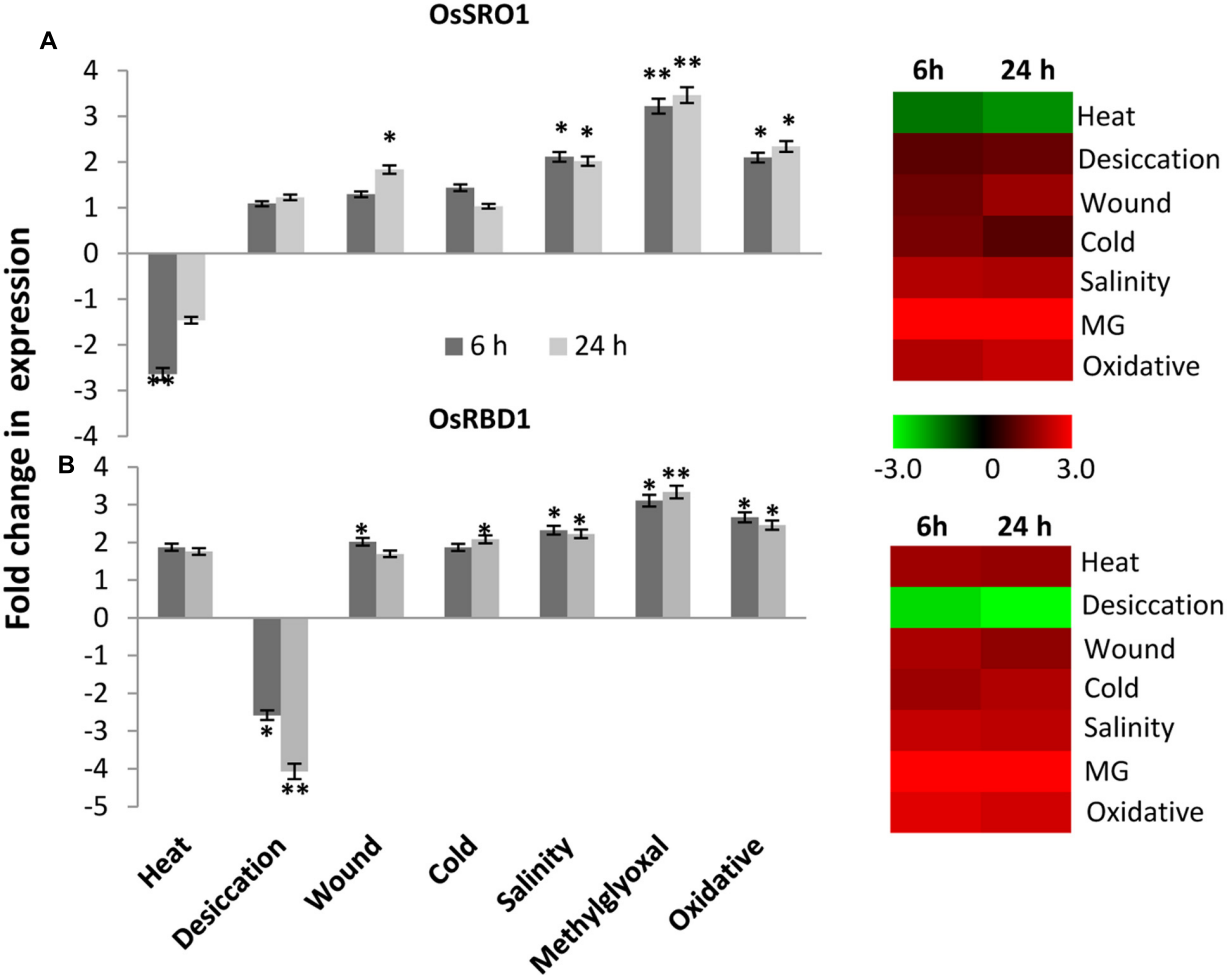
**Expression analysis of **(A)***OsSRO1a* and **(B)***OsRBD1* in response to various abiotic stresses.** Analysis was done using cDNA prepared from shoot tissues of 12 days-old seedlings at control conditions or seedlings subjected to heat (42°C), desiccation (air dry), wound (mechanical injury), cold (4°C), salinity (200 mM NaCl), methylglyoxal (5 mM MG) and oxidative stress (5 mM H_2_O_2_) for 6 h and 24 h. Corresponding heatmaps were generated using TIGR MeV software package. Color bar represents relative expression values; thereby, green color represents lower expression levels, black represents no change, and red signifies higher expression level. Three replicates of each sample were used. Statistically significant change in transcript levels is indicated (^∗^*P* < 0.05; ^∗∗^*P* < 0.001).

To study the relative expression levels of *OsSRO1a* in response to various stresses, quantitative real-time (qRT) PCR analysis was performed in shoots of rice seedlings (Cultivar IR64). *OsSRO1a* was found to be differentially regulated under stress (**Figure 5A**). *OsSRO1a* expression increased threefold in response to methylglyoxal (MG) treatment (a cytotoxic metabolite whose levels rise in response to abiotic stress) throughout the time course of stress treatments and about twofold up-regulation was observed in response to oxidative and salinity stress. Further, wounding, cold and desiccation stress led to ∼1.5-fold increase in *OsSRO1a* expression. However, *OsSRO1a* was down-regulated throughout following heat stress treatment (**Figure 5A**).

Stress-induced expression profile of *OsRBD1* was also examined in order to explore the significance of *OsSRO1a*-*OsRBD1* interactions in stress. For this, qRT-PCR was performed using same set of cDNA as used for *OsSRO1a* expression profiling. Upon comparison, a co-regulation in *OsSRO1a* and *OsRBD1* expression was observed in response to various stress treatments. Like *OsSRO1a*, *OsRBD1* was also found to be up-regulated under most of the stress treatments (**Figure 5B**). About threefold increase in *OsRBD1* levels was observed in response to MG treatment while oxidative stress led to ∼2 fold up-regulation in its expression. In addition, a positive co-regulation in *OsSRO1a* and *OsRBD1* expression was also observed in response to wounding and salinity stress. However, unlike *OsSRO1a* which showed reduced expression on exposure to high temperature, *OsRBD1* expression increased under such condition. In response to desiccation stress, *OsRBD1* transcript levels declined at both 6 h and 24 h (**Figure 5B**).

### OsSRO1a-OsRBD1 Interaction Imparts Stress Tolerance in Yeast

**FIGURE 6:**
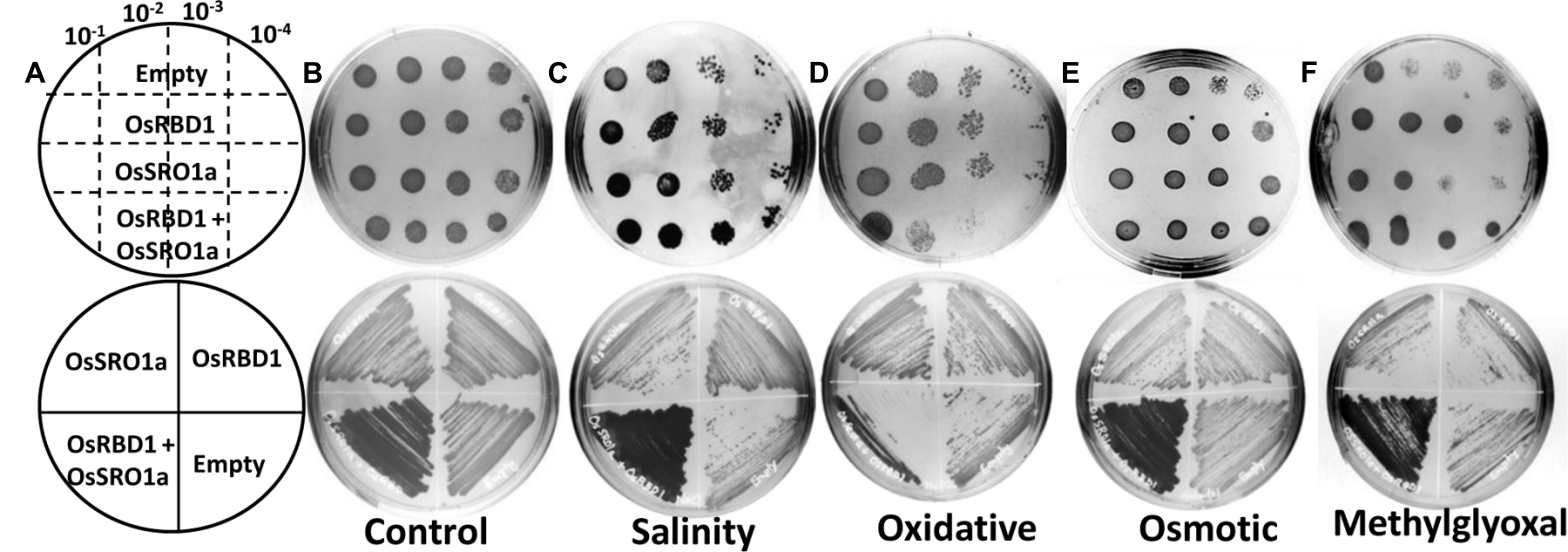
**Interaction between OsSRO1a and OsRBD1 provides stress tolerance to *Saccharomyces cerevisiae*.** **(A)** Schematic depiction of various constructs used for spotting serial dilutions (top panel) and streaking (bottom panel) assays. Comparison of growth and stress tolerance of individually transformed OsSRO1a and OsRBD1 constructs and co-transformed cells containing both the constructs under **(B)** non-stress (control) conditions, **(C)** salinity (1.2 M NaCl), **(D)** oxidative (10 mM H_2_O_2_), **(E)** osmotic (1 M mannitol), and **(F)** methylglyoxal (8 mM) treatments. Empty vector was also transformed and used as control. Top panel in each case represents spotting of serially diluted cultures of the constructs used in the study and bottom panel shows growth after streaking the same constructs. Three clones of each construct were used and the experiment was repeated thrice.

To explore the significance of *OsSRO1a*-*OsRBD1* interactions in stress response, *OsSRO1a* and *OsRBD1* cDNA were cloned in pYES2 vector and transformed, either individually or together, in yeast BY4741 strain. Empty vector (pYES2) was used as control. The positive colonies were confirmed by colony PCR and spotted following serial dilution on solid YPD-agar medium or YPD-agar medium supplemented with stress inducing agents (**Figures 6A–F**). Growth pattern was also compared following streaking the constructs on control and stress media. It was observed that co-transformed constructs containing both *OsSRO1a* and *OsRBD1* genes could grow better in response to salinity and MG treatment as compared to the empty vector or separately transformed *OsSRO1a* and *OsRBD1* constructs (**Figures 6C,F**). However, growth of interacting clones was not significantly different from the individually transformed OsSRO1a and OsRBD1 constructs in case of osmotic stress (**Figure 6E**) but severally hampered in case of oxidative stress (**Figure 6D**), though single clones or empty vector could survive well under similar conditions.

## Discussion

SRO proteins regulate abiotic stress-related and developmental responses by interacting with various transcription factors. In the present study, we report a novel interacting partner of OsSRO1a protein, i.e., an RNA binding domain-containing protein OsRBD1 from rice, which interacts with OsSRO1a through its RRM. OsSRO1a, in addition to poly (ADP-ribose) polymerase domain (PARP), possesses a conserved C-terminal RST domain through which all interactions are mediated; and a WWE domain, present in several RCD1 and SRO proteins. The combination of PARP-RST domain is specific to plants and RST domain-bearing C-terminus sequence of SRO proteins is suggested to be critical for interaction with several, mostly plant specific transcription factors (Jaspers et al., 2009). As reported by various groups, our results also suggest several interacting partners of OsSRO1a in rice, most of which are either transcription factors or related to stress response such as, universal stress domain containing protein (USPs), which is known to play a key role in ethylene mediated stress response in rice (Sauter et al., 2002); a cold acclimation protein WCOR413 involved in low temperature stress (Allard et al., 1998); ubiquitin conjugating enzymes involved in ubiquitination, and many more.

Among several interacting partners of OsSRO1a, we studied detailed interaction with an RNA binding domain-containing protein, OsRBD1, being predominantly present among all the colonies screened. We found that both OsSRO1a and OsRBD1 were nucleus-localized and showed interaction in nucleus. OsRBD1 shows 86% amino acid sequence similarity to ALY2 (AT5G02530) from *Arabidopsis* (Uhrig et al., 2004) and about 46% similarity with YRA1 from yeast (Sträßer and Hurt, 2000), which are export factors playing a key role in splicing-coupled RNA transport from the nucleus and also in transcriptional activation. The RBD proteins, in general, comprise a large family in rice and are recognized as key regulatory factors in the post-transcriptional regulation of gene expression in eukaryotes (Li et al., 2014), and are also found to be actively involved in stress response as well as in development and growth of plants (Teotia and Lamb, 2009). In fact, regulatory mechanisms such as RNA synthesis, processing, transport, translation, storage, stability, and degradation are emerging as important processes involved in the manipulation of cellular responses to stress (Ambrosone et al., 2012).

Our results suggest that OsSRO1a may interact through its RST domain with the RRM1 domain of OsRBD1. Previously, it has been shown that RRM domain of ALY2, the closest *Arabidopsis* homolog of OsRBD1 is central for its interaction with P19 protein of tomato bushy stunt virus in yeast (Uhrig et al., 2004). RRM1 domains possess both RNA and protein binding abilities, and thus, binding of OsSRO1a at the RRM1 domain may alter its RNA binding capacity or its interaction with other proteins, thereby contributing to a mode of regulation of OsRBD1 function. Although function of RNA binding proteins (RBPs) is not characterized in detail, it has been suggested that some members may play a role in stress response, as their mRNA levels are reported to increase following exposure to various stresses (Ambrosone et al., 2012). For instance, *OsDEG10* predicted to encode a small RBP, is strongly induced under several abiotic stress treatments such as, high light, anoxia, salinity, ABA, methyl viologen, and cold; and *OsDEG10* RNAi transgenic plants are more sensitive to high light and cold stresses as compared to wild type plants (Park et al., 2009). Further, an RBP AtRGGA from *Arabidopsis* has also been shown to regulate tolerance to salt and drought stress (Ambrosone et al., 2015). The plants over-expressing *AtRGGA* gene were found to exhibit greater tolerance to ABA and salt stress on plates and in soil and accumulated lower levels of proline when exposed to drought stress. However, molecular mechanisms of how RBPs contribute to plant responses under different stresses are largely unknown.

In order to investigate the significance of OsSRO1a and OsRBD1 interaction in stress, we analyzed the transcriptional profile of both these genes in response to various abiotic stress factors. A positive correlation between *OsSRO1a* and *OsRBD1* expression was seen in response to various stresses such as, low temperature, salinity, wounding, and oxidative stress along with methylglyoxal treatment. However, in case of high temperature and desiccation conditions, *OsSRO1a* and *OsRBD1* expression was negatively correlated. SRO1 is known to function in various stress conditions either in a positive or negative fashion, thereby acting as a regulator of gene expression (Ahlfors et al., 2004; Fujibe et al., 2004; Katiyar-Agarwal et al., 2006; Vainonen et al., 2012). In *Arabidopsis*, RCD1 protein levels are reported to reduce significantly after 30 min of heat shock, whereas its interacting partner DREB2A which is known to be involved in transient and rapid response to heat stress but gradual response to salt and drought stress, was found to be stabilized after 1 h of heat shock. This opposite nature of protein accumulation suggests a negative impact of RCD1–DREB2A interaction, implying that RCD1 may be involved in targeting DREB2A for degradation (Vainonen et al., 2012). Hence, a similar regulation is possible even in case of OsSRO1a and OsRBD1 interaction as suggested by a negative correlation in their expression levels under heat stress.

Though role of *OsRBD1* and its counterparts in other organisms has not been earlier investigated in stress response, yet it has been clearly demonstrated that loss of *YRA1*, an yeast homolog of *OsRBD1* gene results in impaired nuclear poly(A)^+^ RNA export at restrictive growth conditions, with *YRA1* null mutants being non-viable (Sträßer and Hurt, 2000). Yra1p, an intranuclear protein contains an RRM domain and shows *in vitro* RNA–RNA annealing activity. In addition, it is able to directly bind Mex67p, a nuclear mRNA export factor and is considered to be essential for nuclear export of mRNA. Further, YRA1 is known to interact with several other proteins involved in RNA metabolism, some of which are mutually exclusive interactions (Kelly and Corbett, 2009). In view of its role in other organisms, we can speculate that increased *OsRBD1* expression under stress and its interaction with multi-stress inducible *OsSRO1a* at the central RRM1 domain may selectively regulate OsRBD1 protein’s RNA binding ability and hence, nuclear RNA stability and export, or may even modify its transcriptional co-activation ability, thereby regulating protein synthesis in the long run. But this hypothesis needs to be experimentally validated.

In order to investigate the significance of such interaction and its effect on stress response, we checked the growth pattern of yeast strains transformed with *OsSRO1a* and *OsRBD1* under various stresses. The results suggested enhanced tolerance of yeast cells co-transformed with *OsSRO1a* and *OsRBD1* to various stresses such as, salinity and methylglyoxal stress. It has been earlier shown that OsSRO1 and RBDs play a crucial role in salt tolerance (Katiyar-Agarwal et al., 2006; Ambrosone et al., 2015). Further, in response to osmotic stress, the growth of yeast cells co-transformed with *OsSRO1a* and *OsRBD1* was not found to be significantly better than the individually transformed *OsSRO1* or *OsRBD1* or empty vector. Few reports on RBDs however, suggest that these proteins are not actively involved in response to osmotic stress conditions (Kim et al., 2007a,b). For instance, an RNA binding domain-containing protein, AtGRP2 from *Arabidopsis*, has been shown not to affect seed germination under osmotic stress but accelerates seed germination and seedling growth under cold stress (Kim et al., 2007a). In fact, loss of both *rcd1* and *sro1* in *Arabidopsis* results in increased resistance to osmotic stress (Teotia and Lamb, 2009). Here, we could see that though the growth of OsSRO1a over-expressing yeast cells was not hampered but even its interaction with OsRBD1 did not result in any growth difference. Furthermore, oxidative stress resulted in a negative response that is, decreased stress tolerance of the co-transformed yeast strain despite a positive correlation in expression between the two interacting partners in oxidative stress, thereby depicting differential regulatory effect of *OsSRO1a* binding at the RRM1 domain of *OsRBD1.* The reduced oxidative stress tolerance of OsSRO1a upon interaction with OsRBD1 finds a support from the existing literature. For instance, a co-dominant *rcd1* mutant has been shown to accumulate superoxide ions and transient spreading lesions in response to ozone and superoxides but not H_2_O_2_ (Overmyer et al., 2000). Hydrogen peroxide at concentration as high as 10 mM was found to be ineffective in inducing cell death in the *rcd1* mutant (Overmyer et al., 2000). On similar lines, it has been also shown that *rcd1* mutant of *Arabidopsis* is more resistant to methyl viologen but very sensitive to ozone as compared to wild type (Fujibe et al., 2006), suggesting that *RCD1* shows a differential response to different elicitors of oxidative stress response. Further, *OsSRO1c*-overexpressing rice has been found to be highly sensitive to oxidative stress but shows enhanced tolerance toward drought conditions since it promotes H_2_O_2_-mediated stomatal closure (You et al., 2013). Though role of SRO proteins has been described in different stresses, such as salinity (Katiyar-Agarwal et al., 2006), dehydration (Vainonen et al., 2012), and oxidative stress (Fujibe et al., 2004; Jaspers et al., 2009), this study might demonstrate another aspect of SRO-mediated regulation by affecting mRNA stability through its interaction with OsRBD1, a close homolog of RNA export factors (Uhrig et al., 2004).

Taken together, this study describes a novel interacting partner of SRO proteins, i.e., OsRBD1, which interacts through its RRM1 motif with the RST domain of OsSRO1a. Further, we have shown that this interaction has implications in stress response in yeast. However, detailed studies are needed to functionally validate such role in plants.

## Author Contributions

SS and CK performed the experiments and wrote the manuscript. SLS-P and SKS conceived the idea, designed the experiments and edited the manuscript. All the authors approved the final manuscript.

## Conflict of Interest Statement

The authors declare that the research was conducted in the absence of any commercial or financial relationships that could be construed as a potential conflict of interest.

## Footnotes

1 http://rice.plantbiology.msu.edu/index.shtml

2 http://www.ncbi.nlm.nih.gov/geo/

3 http://pfam.xfam.org/

4 http://www.ricearray.org/

5 http://bioinfo.ggc.org/bindn/
