# Supplementary material for: OsSRO1a Interacts with RNA Binding Domain-Containing Protein (OsRBD1) and Functions in Abiotic Stress Tolerance in Yeast
Source: Front Plant Sci. 2016 Feb 3;7:62. doi: 10.3389/fpls.2016.00062 (PMC4737904; doi:10.3389/fpls.2016.00062)
Supplement: Supplementary file 1 [file Table_1.DOCX]

**Supplementary Table S1**. List of primers used in the study.

| Primer name | Nucleotide sequence (5’-3’) |
| --- | --- |
| SRO1a_FL_Fwd | CGGGATCC ATGGCTGCGATGAACGAAAAG |
| SRO1a_FL_Rev | CGGGATCCTTAGCCATCATCTTGGCCCACGTGTT |
| SRO1a_N_Fwd | CGGGATCCATGGCTGCGATGAACGAAAAG |
| SRO1a_N_Rev | CGGGATCCAGATCTGCAGGGAATAGGCCAT |
| SRO1a_pARP_Fwd | CGGGATCCATGCTACCACAAGATAAGCTG |
| SRO1_pARP_Rev | CGGGATCCCTATTCCACTTTATCTCCTAACAT |
| SRO1a_C_Fwd | CGGGATCCATGAGGGCTCCAAGCACGCCTT |
| SRO1a_C_Rev | CGGGATCCTTAGCCATCATCTTGGCCCACGTGTT |
| SRO1a_ RT_Fwd | TTTCCGATGCGAAATGATGATT |
| SRO1a_ RT_Rev | GGCGAAACAATAACATTAATCA |
| RBD1_FL_Fwd | GGAATTCATGGCGGCGGACTCGCTGCT |
| RBD1_FL_Rev | ACGCGTCGACCTAGCTCATTTGCATTCCTT |
| RBD1_N_Fwd | GGAATTCATGGCGGCGGACTCGCTGCT |
| RBD1_N_Rev | ACGCGTCGACCTACTTGGTGCCGGTCTCGA |
| RBD1_RRM1_Fwd | GGAATTCATGCTCTACATCTCCAACCTCGA |
| RBD1_RRM1_Rev | ACGCGTCGACCTATTTCATAGGTTTGCCAT |
| RBD1_C_Fwd | GGAATTCCTTGAGCTCATTGGTATA |
| RBD1_C_Rev | ACGCGTCGAC CTAGCTCATTTGCATTCCTT |
| RBD1_RT_Fwd | AAGCTAGCTCATAATATGTAA |
| RBD1_RT_Rev | AATCAATTCAATCCTCTTGCCA |
