# Supplementary material for: OsSRO1a Interacts with RNA Binding Domain-Containing Protein (OsRBD1) and Functions in Abiotic Stress Tolerance in Yeast
Source: Front Plant Sci. 2016 Feb 3;7:62. doi: 10.3389/fpls.2016.00062 (PMC4737904; doi:10.3389/fpls.2016.00062)
Supplement: Supplementary file 2 [file Table_2.DOCX]

**Supplementary Table S2**. Interacting partners of OsSRO1a found through yeast two hybrid assay.

| **Clone no.** | **Locus ID** | **Homology (%)** | **Description** |
| --- | --- | --- | --- |
| 1 | LOC_Os01g42860 | 97 | inhibitor I family protein, putative, expressed |
| 3 | LOC_Os01g01010 | 74 | TBC domain containing protein, expressed |
| 5 | LOC_Os07g01150 | 93 | fatty acid hydroxylase, putative, expressed |
| 8 | LOC_Os12g01010 | 99 | RNA recognition motif containing protein, putative, expressed |
| 11 | LOC_Os03g06730 | 56 | plant protein of unknown function domain containing protein, expressed |
| 12 | LOC_Os06g51220 | 99 | HMG1/2, putative, expressed |
| 14 | LOC_Os11g43900 | 79 | translationally-controlled tumor protein, putative, expressed |
| 15 | LOC_Os08g39090 | 89 | coiled-coil domain containing 49, putative, expressed |
| 18 | LOC_Os02g13950 | 66 | NHL repeat-containing protein, putative, expressed |
| 19 | LOC_Os12g36180 | 96 | auxilin, putative, expressed |
| 20 | LOC_Os01g42860 | 77 | inhibitor I family protein, putative, expressed |
| 21 | LOC_Os05g23240 | 65 | CAX-interacting protein 4, putative, expressed |
| 22 | LOC_Os09g17740 | 88 | chlorophyll A-B binding protein, putative, expressed |
| 24 | LOC_Os12g19381 | 99 | ribulose bisphosphate carboxylase small chain, chloroplast precursor, putative, expressed |
| 25 | LOC_Os01g19820 | 100 | universal stress protein domain containing protein, putative, expressed |
| 29 | LOC_Os05g01020 | 90 | transcriptional repressor, putative, expressed |
| 34 | LOC_Os04g57220 | 95 | ubiquitin-conjugating enzyme, putative, expressed |
| 35 | LOC_Os10g35480 | 88 | lanC-like protein 2, putative, expressed |
| 39 | LOC_Os08g38170 | 99 | methyladenine glycosylase, putative, expressed |
| 41 | LOC_Os06g07300 | 77 | jacalin-like lectin domain containing protein, expressed |
| 44 | LOC_Os11g47809 | 88 | metallothionein, putative, expressed |
| 45 | LOC_Os06g01850 | 96 | ferredoxin-NADP reductase, chloroplast precursor, putative, expressed |
| 47 | LOC_Os11g08300 | 91 | aldehyde dehydrogenase, putative, expressed |
| 48 | LOC_Os03g32170 | 97 | NAD dependent epimerase/dehydratase family protein, putative, expressed |
| 53 | LOC_Os06g01060 | 100 | retrotransposon protein, putative, Ty3-gypsy subclass, expressed |
| 65 | LOC_Os02g01010 | 100 | OsPDIL1-4 protein disulfide isomerase PDIL1-4, expressed |
| 66 | LOC_Os03g27370 | 99 | phospholipase D, putative, expressed |
